# Solubilizing Metal–Organic Frameworks for an *In Situ* IR-SEC Study of a CO_2_ Reduction Catalyst

**DOI:** 10.1021/acsami.2c20157

**Published:** 2023-03-21

**Authors:** Wenmiao Chen, Wai Yip Fan, Muhammad Sohail, Sherzod T. Madrahimov, Ashfaq A. Bengali

**Affiliations:** † Division of Arts and Sciences, Texas A&M University Qatar, PO Box 23874, Education City, Doha, Qatar; ‡ Department of Chemistry, Texas A&M University, Galveston, Texas 77553, United States; § National University of Singapore, Singapore 119077, Singapore

**Keywords:** catalyst, electrocatalyst, MOFs, carbon dioxide reduction, infrared spectroelectrochemistry (IR-SEC)

## Abstract

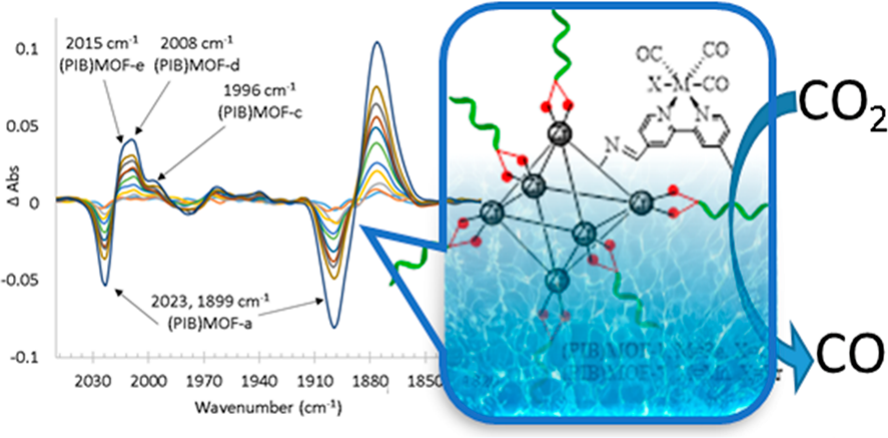

Metal–organic frameworks (MOFs) are typically assembled by bridging metal centers with organic linkers for various applications, including providing robust support for heterogeneous catalysts for CO_2_ reduction. In this study, we have demonstrated the solubilization of a MOF tethered to a CO_2_-reducing electrocatalyst and studied its fundamental electrochemistry in THF solvent using infrared spectroelectrochemistry (IR-SEC). The fundamental electrochemical properties of this immobilized catalyst were compared to that of its homogeneous counterpart. This approach provides a foundation for future experimental studies to bridge the gap between homogeneous and heterogeneous electrocatalysis.

## Introduction

Heterogeneous catalysts are primarily used in industrial-scale applications due to their stability, facile scalability, and activity.^1−3^ However, the discovery and optimization of these catalysts rely mostly on high-throughput screening, and a lack of mechanistic understanding typically hinders the rational design of highly selective heterogeneous catalysts.^2,3^ By comparison, molecular-engineered homogeneous catalysts are mechanistically well-understood.^2^ To bridge the gap, molecular complexes are typically immobilized onto a solid surface to provide mechanistic insight into the activity of the resulting species, aiding in the development of catalysts that are both efficient and selective.^2,4^

Recently, metal–organic frameworks (MOFs) have been used as supports for well-defined transition-metal-based complexes, thereby addressing a disadvantage of heterogeneous catalysts, namely, the lack of information about the molecular structure.^5−10^ Taking advantage of these opportunities, a variety of molecular catalysts have been grafted onto MOFs for various applications such as CO_2_ reduction.^11−16^ In some cases, anchoring these complexes to a MOF support results in better catalytic activity than the molecular system.^10,17,18^ For example, the (bipy)Re(CO)_3_X and (bipy)Mn(CO)_3_X [bipy = 2,2′-bipyridine, X = Cl^–^, Br^–^] complexes are among the most well-studied photo- and electrocatalysts for CO_2_ reduction and have recently been grafted onto MOFs to avoid deactivation due to dimerization which impedes catalytic activity.^11,19−23^ These MOFs also offer the possibility of CO_2_ absorption, which may enhance catalytic activity by increasing the effective substrate concentration at active sites.^16,22,24^

However, because MOFs are not soluble and therefore not accessible to traditional condensed-phase transmission spectroscopies, it is difficult to identify, characterize, and study the reactivity of the relevant intermediate species generated in these reactions. This limitation makes it challenging to obtain mechanistic information about the reactions and impedes the application of a rational design process to develop more efficient catalysts. Recently, we established that tethering polar and nonpolar oligomers to the MOF surface generates MOF nanoparticles that are soluble in both polar and nonpolar solvents and can function as supports for transition metal catalysts that are observable by transmission IR spectroscopy.^25^ This discovery opens the way to investigate the operational mechanism of a number of catalytic processes mediated by MOF-immobilized transition metal catalysts using traditional spectroscopic methods to monitor the reaction progress and identify relevant intermediates.

The technique of infrared spectroelectrochemistry (IR-SEC) has played a key role in elucidating the mechanism of CO_2_ reduction by the molecular (bipy)Re(CO)_3_X and (bipy)Mn(CO)_3_X complexes.^26−30^ In the present work, we extend these studies to investigate the analogous electrochemistry of the solubilized MOF-supported (bipy)Re(CO)_3_Cl catalyst. Solubilization of the MOF particles in THF was achieved by grafting polyisobutylene (PIB) oligomers onto the MOF surface, thereby enabling *in situ* acquisition of solution-state IR spectra under applied potentials. The basic electrochemistry of this catalytic system was investigated and found to mimic the behavior of the homogeneous molecular system with some differences. Such experiments were previously inaccessible due to the insolubility of MOFs and therefore serve as a first example of the application of this technique toward an in-solution study of MOF-immobilized transition-metal complexes for CO_2_ reduction. It is hoped that this proof-of-concept study can be further leveraged to gain mechanistic insight into reactions catalyzed by single-molecule heterogeneous complexes tethered to MOF surfaces.

Solubilization of nanoparticles in organic solvents can be achieved by grafting polyethene glycol (PEG) and polyisobutylene (PIB) oligomers with functionalized −COOH, −Si(OR)_3_, and −PO(OH)_2_ groups to generate phase-separatable and recyclable catalysts.^31,32^ Similarly, monomers and oligomers (PIB, PEG) containing different functional end groups can also be tethered to MOFs by coordination with the metal-oxo nodes.^17,33−35^

## Results and Discussion

**Figure 1:**
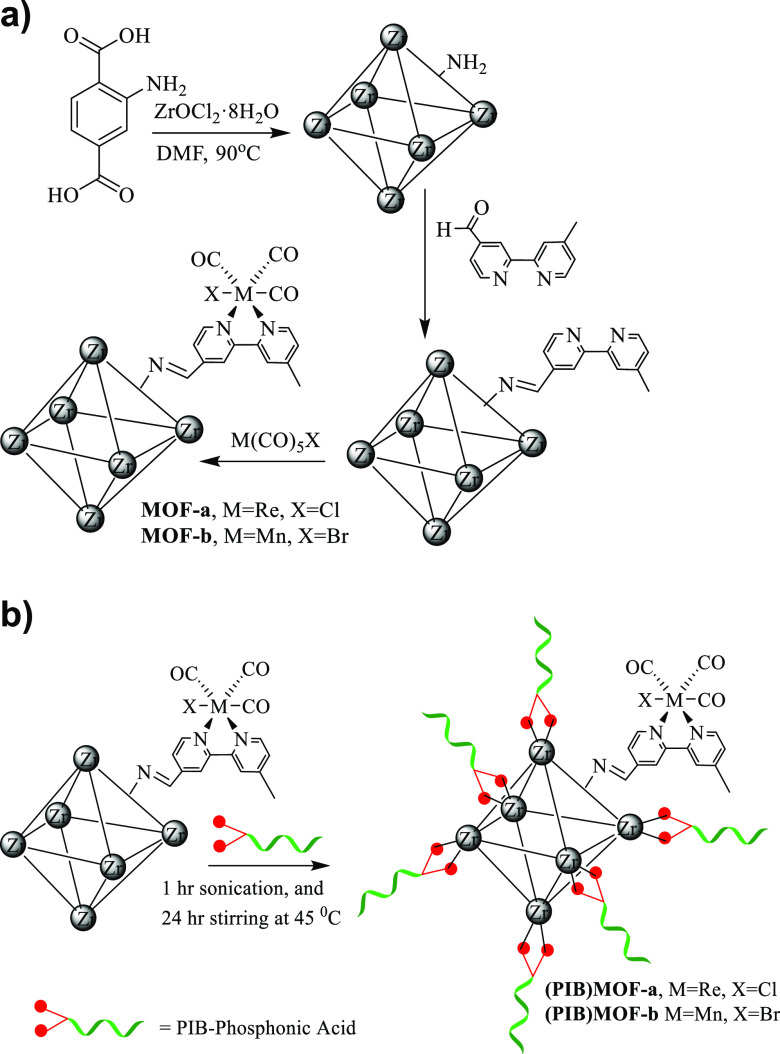
(A) Synthetic procedure for immobilization of catalyst on **UiO66-NH**_**2**_ to form **MOF-a** and **MOF-b**. (B) Synthetic procedure for the grafting of PIB onto the metal-oxo nodes of **MOF-a** and **MOF-b** to form **(PIB)MOF-a** and **(PIB)MOF-b**, respectively

Recently, we reported a method to solubilize an azide-functionalized MOF (UiO66-N_3_) with PIB. An alkyne bipyridine Re carbonyl complex was tethered to the MOF surface with catalytic click chemistry.^25^ Simplifying this approach, in this study, the UiO66-NH_2_ MOF was functionalized with aldehyde derivatives of bipyridine by an uncatalyzed condensation reaction under sonication and reflux conditions.^15^ Under these conditions, a toluene suspension of this MOF in the presence of M(CO)_5_X [M = Re, Mn, X = Cl and Br] resulted in the immobilization of the (bipy)M(CO)_3_X complex on the MOF surface (Figure 1A). Subsequently, phosphonic-acid-functionalized PIB was grafted onto the resulting MOF by utilizing its metal-oxo nodes (Figure 1B, see SI for details).

The presence of metal carbonyl complexes on the MOF surface was confirmed using a variety of techniques as reported previously, including IR spectroscopy, powder X-ray diffraction (PXRD), scanning electron microscopy (SEM), energy-dispersive X-ray spectroscopy (EDX) mapping, and TGA (Figures S2–6).^25^ The EDX analysis showed a uniform distribution of the immobilized complexes throughout the surface of these MOFs (Figures S4–5). The results of PXRD and SEM analyses verified the conservation of the crystalline structure of the materials during the condensation reaction (Figures S2–3). A solid-state IR spectrum of both UiO66-(bipy)Re(CO)_3_Cl (**MOF-a**) and **(PIB)MOF-a** showed peaks at 2031 cm^–1^ and a broad peak at 1933 cm^–1^ (Figure S7). In the solution state (Figure S9), the corresponding peaks for **(PIB)MOF-a** were observed at 2023 and 1899 cm^–1^ (broad). These absorbances were assigned to the Re-bound terminal CO groups, and their positions are comparable to those observed for the analogous molecular complex (Figure S8).^30^ Similarly, the solid-state IR spectrum of UiO66-(bipy)Mn(CO)_3_Br (**MOF-b**) displayed peaks at 2031 cm^–1^ and a broad peak at 1933 cm^–1^ (Figure S10). Because of its greater stability, IR-SEC experiments were conducted only on the rhenium system, **(PIB)MOF-a**.

**Figure 2:**
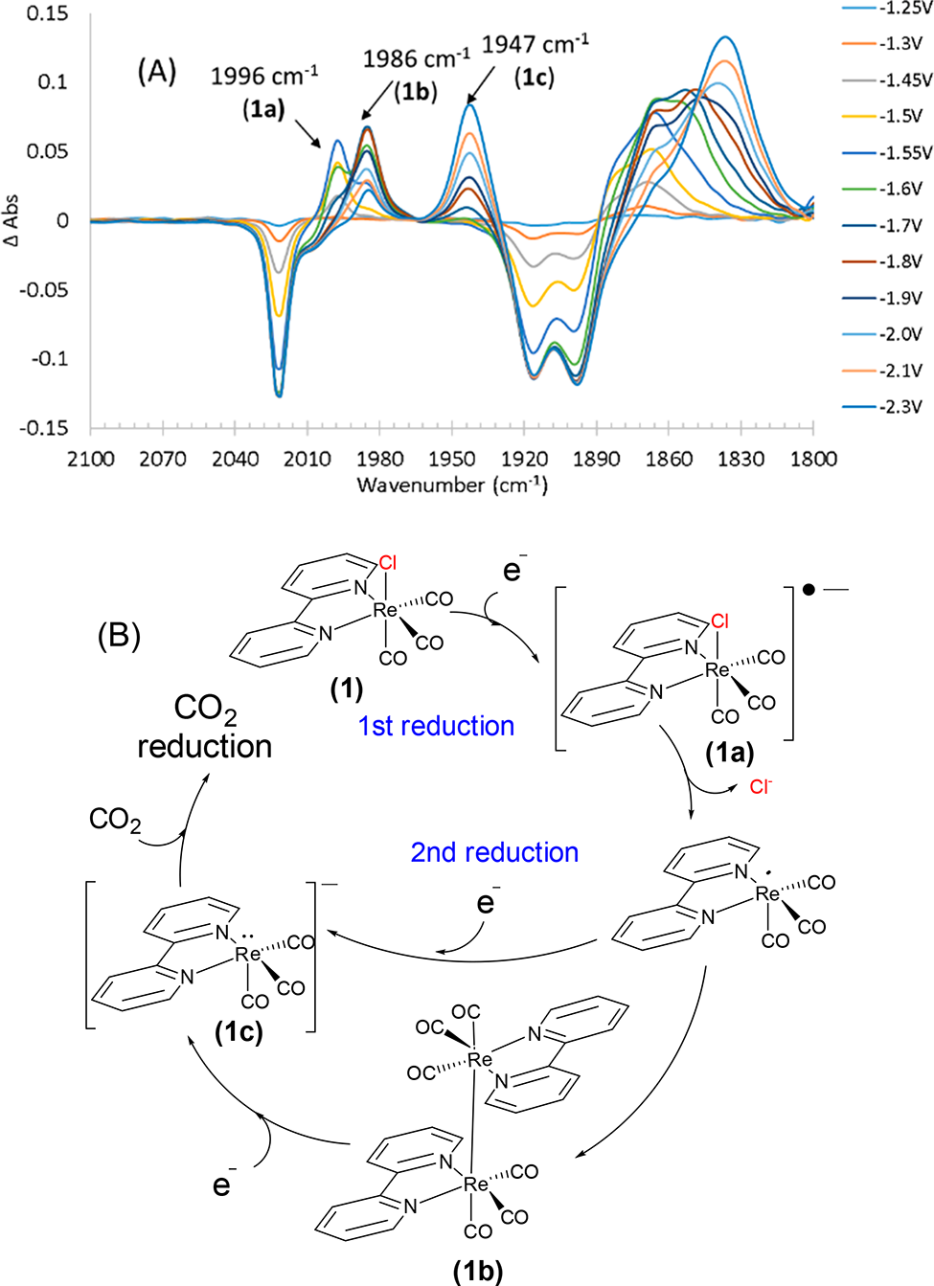
(A) Difference FTIR spectra obtained upon electrolysis of **1** over a voltage range of −1.25 to −2.50 V at a scan rate of 100 mV/s. The reference IR spectrum is of the solution before application of a potential difference. (B) Intermediates observed during the electrolysis of **1** in THF (adapted from ref (9) c). While this experiment was conducted in the absence of CO_2_, intermediate **1c** has been identified previously as the catalytically active species in CO_2_ reduction.

To compare the condensed-phase electrochemistry of the MOF-immobilized and molecular rhenium carbonyl complexes, initial investigations focused on (bipy)Re(CO)_3_Cl (**1**). This complex was subjected to an IR-SEC study in THF solvent to identify and establish the spectral features of important electrocatalytic intermediates. The observed spectral changes were consistent with those reported in the literature and are shown in Figure 2A. Consistent with the work of Kubarych and co-workers, the intermediates detected are shown in Figure 2B.^28^ In the present study, as the voltage was scanned at the rate of 2 mV/s, covering a range from 0 to −2.5 V (vs the Fc/Fc^+^ couple), the characteristic CO peaks of the parent complex **1** in THF (2023, 1918, and 1896 cm^–1^) decreased in intensity, while growth of peaks assigned to the first reduced species [(bipy)Re(CO)_3_Cl]^−^* (**1a**) began to appear at 1996, 1883, and 1868 cm^–1^. As the voltage was further decreased, signature peaks of the dimer species (bipy)_2_Re_2_(CO)_6_ (**1b**) appeared at 1986 cm^–1^, 1950 cm^–1^, 1888 cm^–1^, and 1854 cm^–1^ followed by the generation of the catalytically active second reduced species [(bipy)Re(CO)_3_]^−^ (**1c**) with CO stretching absorbances at 1947 and 1843 cm^–1^.

**Figure 3:**
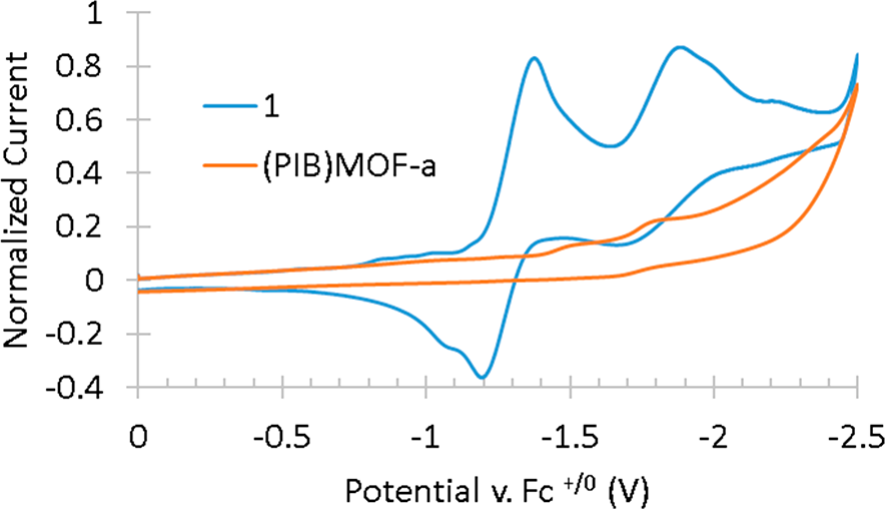
CV of **1** and **(PIB)MOF-a** in THF solvent. All measurements were recorded at 100 mV/s, under argon in THF with 0.1 M TBAPF_6_ supporting electrolyte (WE = glassy carbon, CE = Pt wire, RE = Ag/AgCl, and Fc^+/0^ as internal reference standard).

The fundamental electrochemistry of **(PIB)MOF-a** was studied in a manner similar to that for complex **1**. Thus, **(PIB)MOF-a** was dissolved in a THF solution containing 0.1 M TBAPF_6_ as the electrolyte and the voltage scanned at a rate of 2 mV/s covering the range from 0 to −2.0 V. The cyclic voltammograms (CVs) of **1** and **(PIB)MOF-a** are shown in Figure 3. Consistent with the literature, the first and second reduction waves for **1** are observed at −1.38 V and −1.88 V, respectively.^27^ The reduction potentials for **(PIB)MOF-a** are shifted slightly from those of **1**, appearing at −1.50 V and −1.78 V, respectively, and suggest that immobilization of (bipy)Re(CO)_3_Cl on the MOF surface does not significantly affect the reduction potentials of this complex. Unlike **1**, the first reduction at −1.50 V does not appear to be reversible in the case of **(PIB)MOF-a** which may be due to decreased solubility of the reduced complexes.

**Figure 4:**
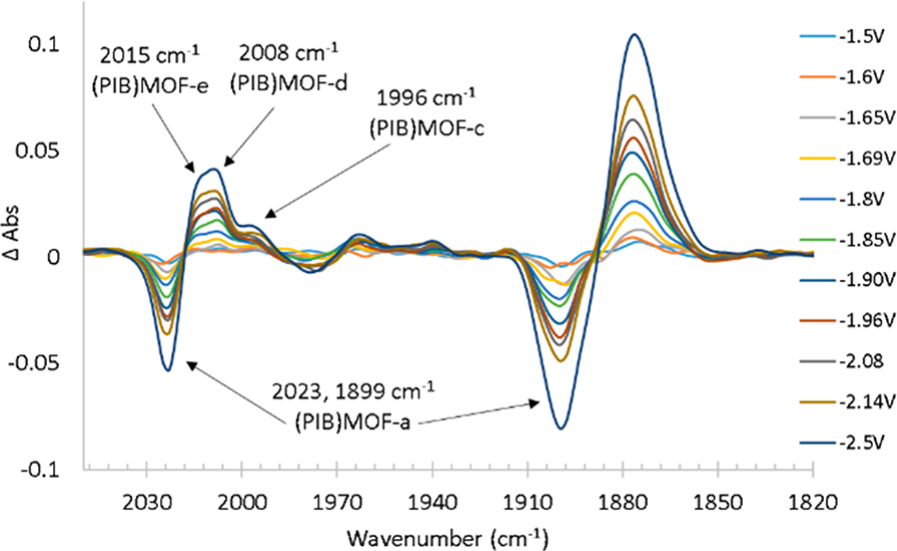
Observed difference IR-SEC spectra obtained upon electrolysis of **(PIB)MOF-a** in THF solvent at scan rate of 2 mV/s. The reference spectrum is of the solution before application of a potential difference.

As shown in Figure 4, the IR-SEC spectra for **(PIB)MOF-a** are quite different from those obtained upon electrolysis of **1**. In general, the CO stretching absorbances of the immobilized rhenium carbonyl complex in **(PIB)MOF-a** are broad, most likely due to differences in the local environment on the MOF surface. As the voltage is scanned negative, the parent peaks decrease in intensity, and four new peaks are observed. Similar to **1**, the broad CO stretching band from 1888 to 1850 cm^–1^ most likely includes the *E* symmetry stretches of several electrochemically generated species with a *fac*-[Re(CO)_3_] geometry, and the lack of spectral resolution in this region does not assist in determining the identity of these intermediates. However, the companion A_1_ stretches observed in the high cm^–1^ region are somewhat better resolved and are more amenable to interpretation. Peaks at 1996, 2008, and 2015 cm^–1^ (overlapped) are observed corresponding to the generation of three possible intermediates.

**Figure 5:**
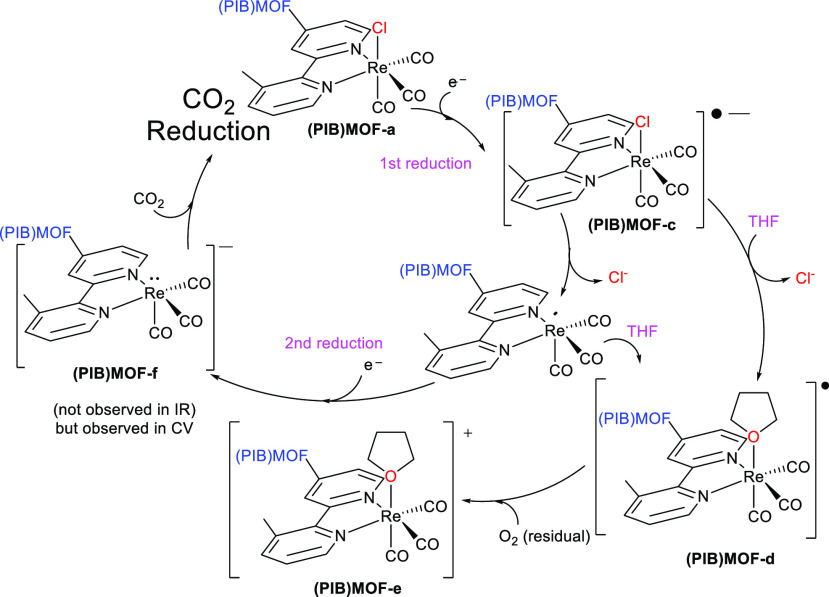
Proposed initial mechanism of the **(PIB)MOF-a** electrocatalytic reduction cycle in THF. While CO_2_ was not added to the solution, it is shown in this scheme to complete the cycle.

In identifying the species associated with these peaks, we have relied on the reported values of the CO stretching cm^–1^ of intermediates observed upon electrolysis of several (α-diimine)Re(CO)_3_X complexes.^30,36^ This comparison is justified because the position of the CO stretching bands of **1** (2023, 1918, and 1896 cm^–1^) and **(PIB)MOF-a** (2023 and 1899 cm^–1^ (broad)) are similar, suggesting that tethering **1** to the MOF does not dramatically influence the electron density on the Re center. Thus, the peak at 1996 cm^–1^ is assigned to the first reduced species (PIB)MOF-[(bipy)Re(CO)_3_Cl]^−^* **[(PIB)MOF-c**], and those at 2008 and 2015 cm^–1^ are assigned to (PIB)MOF-[(bipy)Re(CO)_3_THF]* **[(PIB)MOF-d]** and (PIB)MOF-[(bipy)Re(CO)_3_THF]^+^**[(PIB)MOF-e]**, respectively. All three complexes have been observed before during the reduction of (α-diimine)Re(CO)_3_Cl complexes in THF. For example, Hartl et al. reported the formation of the THF-substituted radical complex upon reduction of (dpp)Re(CO)_3_Cl [dpp = 2,3-di(2-pyridylpyrazine)], which was generated upon dissociation of Cl^–^ from the initially produced [(dpp)Re(CO)_3_Cl]^−^* species.^30,36^ Notably, the THF radical species was mostly observed in complexes in which dimerization was not detected, presumably due to the steric bulk of the diimine ligand (which would certainly be the case in the present study). Finally, as previously reported for the molecular system, the cationic species **(PIB)MOF-e** may be generated by oxidation of the radical species **(PIB)MOF-d** by residual oxygen in solution.^28^ The proposed reaction scheme upon electrolysis of **(PIB)MOF-a** is shown in Figure 5.

In dramatic contrast to **1**, evidence in the IR-SEC spectra was not obtained for the formation of the analogous dimer or the second reduced species **(PIB)MOF-f** in the electrolysis of **(PIB)MOF-a**. The lack of dimerization can be easily justified since the large steric profile of the MOF would inhibit this reaction, as in the case of other molecular (α-diimine)Re(CO)_3_X complexes.^2,26,30,36^ The failure to observe a CO stretching absorbance consistent with the second reduced species in the MOF system (expected at ∼1950 cm^–1^), even though the CV confirms a second reduction step (Figure 3), is harder to explain. Perhaps an insufficient amount of **(PIB)MOF-f** is formed to be detected by IR spectroscopy, or it is insoluble in THF and is therefore unobservable in solution.

**Figure 6:**
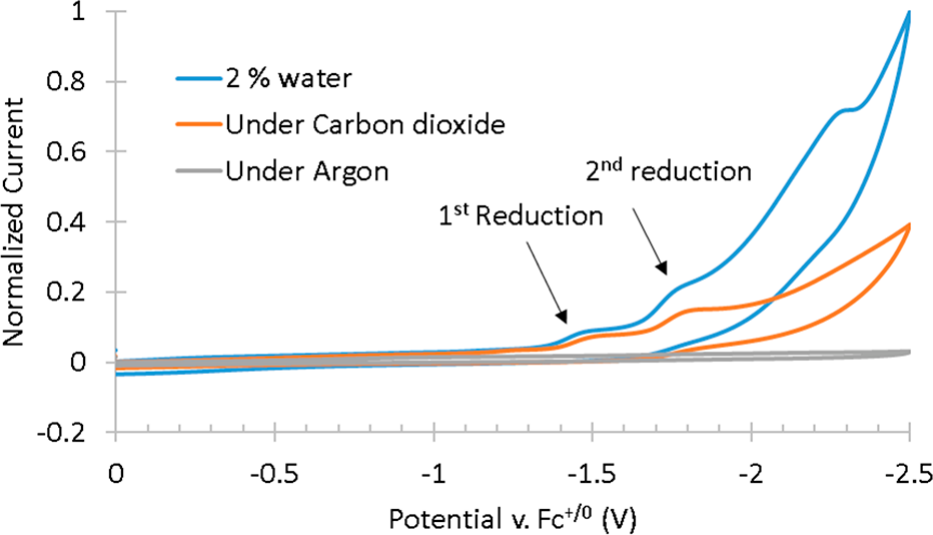
CV of **(PIB)MOF-a** in THF solution in the only Ar, CO_2_, and CO2 and water. Water acts as proton source to complete the reduction of CO_2_. All measurements were recorded at 100 mV/s, under argon in THF with 0.1 M TBAPF_6_ supporting electrolyte (WE = glassy carbon, CE = Pt-wire, RE = Ag/AgCl, and Fc^+/0^ as internal reference standard).

A few preliminary experiments were conducted to determine whether **(PIB)MOF-a** was capable of reducing CO_2_ like its molecular counterpart. The CV of a CO_2_-saturated THF solution of complex **(PIB)MOF-a** shows a slight increase in current at both reduction potentials, which is indicative of catalytic activity. The increase in current at −1.77 V indicates the formation of the second reduced species, which is responsible for CO_2_ reduction (Figure 6).

However, because it is challenging to calculate the concentration of the rhenium complex in the MOF system, a direct comparison of catalytic efficiency between **1** and **(PIB)MOF-a** remains elusive. Further investigations are ongoing to identify and study the reactivity of possible CO_2_ reduction intermediates. Different MOF motifs (e.g., replacement of Zr by Hf) are also being explored.

## Conclusion

This work highlights a new method for solubilizing heterogeneous electrocatalyst-immobilized MOFs in organic solvents for *in situ* spectroscopic studies. We have shown that condensed-phase IR-SEC can be used to identify electrochemically generated intermediates that have previously been difficult to access. It is hoped that this work can be leveraged to perform *in situ* mechanistic studies of single-molecule heterogeneous electrocatalysts using solution-phase analytic techniques.

## Supporting Information Available

Detailed descriptions of synthetic procedures and other experiments as well as analytical data (PDF)
