# Supplementary material for: Solubilizing Metal–Organic Frameworks for an In Situ IR-SEC Study of a CO2 Reduction Catalyst
Source: ACS Appl Mater Interfaces. 2023 Mar 21;15(13):16593–7. doi: 10.1021/acsami.2c20157 (PMC10848202; doi:10.1021/acsami.2c20157)
Supplement: Supplementary file 1 — am2c20157_si_001.pdf [file am2c20157_si_001.pdf]

Supporting Information

Solubilizing MOFs for an *In Situ* IR-SEC study of a CO_2 reduction catalyst.

Wenmiao Chen,[a,b] Wai Yip Fan,[c] Muhammad Sohail,[a,*] Sherzod T. Madrahimov,[a,*] Ashfaq A. Bengali.[a,*]
[a]Texas A&M University Qatar. [b] Texas A&M University. [c] National University of Singapore.

*Correspondence to: ashfaq.bengali@qatar.tamu.edu and sherzod.madrahimov@qatar.tamu.edu

muhammad.sohail@qatar.tamu.edu

Table of content

Contents

1. Materials and methods: 1
   1.1. Materials 1
   1.2. Instrumentation 1
2. Synthetic Methods: 3
   2.1. Synthesis of UiO66-NH_2 MOF: 3
   2.2. Synthesis of UiO66-bpy (MOF-byp): 3
   2.3. Synthesis of **MOF-a** (M= Re, X= Cl) and **MOF-b** (M=Mn, X=Br): 3
   2.4. Synthesis of **(PIB)MOF-a** 3
3. Figures S2 to S9 4
4. References 9

1. Materials and methods:

1.1. Materials

All reactions involving air and moisture sensitive compounds were carried out under a nitrogen atmosphere. Anhydrous tetrahydrofuran (THF), anhydrous toluene, tetrabutylammonium hexafluorophosphate ($TBAPF_6$), anhydrous cyclohexane and anhydrous dichloromethane (DCM) were obtained from Sigma-Aldrich and used as received. UiO66-NH_2 was prepared and activated according to a previously published procedure by Morris et al. [1]. CHO-Bipy was purchased from Alpha Chemicals (China) and used as received. All glassware was oven-dried before use.

All oven-based MOF syntheses were carried out in explosion-proof HERAtherm OMS-100 (Thermo Fisher Scientific, Waltham, MA, USA) ovens that had been pre-heated to a preset temperature. Sonication was carried out with a Fisher Scientific Ultrasonic Cleaner FS60 (Thermo Fisher Scientific, Waltham, MA, USA).

1.2. Instrumentation

Infrared spectra of solid samples was collected using a Perkin-Elmer FTIR (Model Spectrum One) fitted with a Universal ATR unit.

Powder X-Ray Diffraction (XRD) spectra were collected using a Rigaku Ultima IV multipurpose X-ray diffractometer equipped with Cu Ka radiation source and a fixed monochromator. The XRD was operated at 40 KV and 40 mA, and a fixed time scan mode with a 0.02 degree step width and 1 sec/step count time used for data collected from 5 to 90 degrees.

Scanning Electron Microscopy/Energy Dispersive X-Ray Spectroscopy (SEM/EDS) was performed using a FEI Quanta 400 environmental scanning electron microscope. The SEM was operated at a 30 KV acceleration voltage at a working distance of 6.5 mm. An EDAX Apollo EDS system was used for EDS signal collection and analysis. For SEM EDS analysis of **(PIB)MOF-a** , 20 µl of the solution sample was dropped over an aluminum sample holder and dried at room temperature.

An IR OTTLE cell (Frantisek Hartl, University of Reading) was used for the IR-SEC experiments. This cell has Pt-minigrid working and auxiliary electrodes and an Ag wire pseudoreference electrode, which is melt-sealed into a polyethylene spacer (200 µm) with 2 mm thick CaF_2 windows [2]. Voltage scans within the OTTLE cell were carried out using a PalmSens EmStat3 potentiostat. Solution state transmission IR spectra during electrolysis were obtained using a Bruker Vertex 80 FTIR equipped with step-scan and rapid-scan capabilities (2200 to 1750 cm^{-1}) at a CV scan rate of 2mV/sec from -1.2 to -2.5V. All spectra were obtained at 4 cm^{-1} resolution.

For both the IR-SEC and cyclic voltammetry experiments, the solution samples were prepared by dissolving either 50mg or 5mg of **PIB(MOF)-a** and (bpy)Re$(CO)_3$Cl (**1**), respectively, in 5ml of dry THF solvent with 0.1M $TBAPF_6$. For the IR-SEC experiments, the background spectrum of the solution was obtained before the potential scan was initiated.

Cyclic voltammetry of **(PIB)MOF-a** and (bpy)Re$(CO)_3$Cl (**1**) was conducted at a scan rate of 2 mV/s and 100 mV/s, respectively, in dry THF solvent with 0.1M $TBAPF_6$ under nitrogen. The electrochemical cell included a 3mm diameter glassy carbon working electrode, a Pt counter electrode, and an Ag/AgCl reference electrode with internal Fc/Fc^+ as an internal reference [3]. Experiments were purged with nitrogen or CO_2 at saturation by bubbling for 20 minutes before CVs were taken and stirred between successive experiments. Figures S1A and S1B are different views of the experimental setup, and the clarity of the solution, which contains the catalyst, demonstrates the complete solubility of **(PIB)MOF-a** in THF solvent.

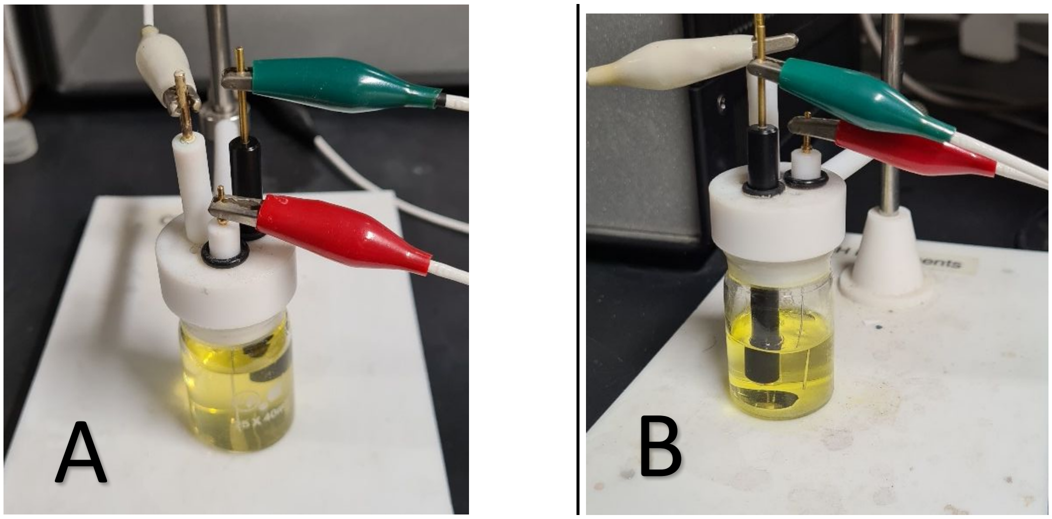

Figure S1: different views of the experimental setup for the CV experiments. The clear solution contains **(PIB)MOF-a** in THF solvent with 0.1 M $TBAPF_6$.

2. Synthetic Methods:

2.1. Synthesis of UIO66-NH_2 MOF:

The yellowish crystalline UiO66-NH_2 MOF was obtained by a solvothermal reaction of amino-triphenyldicarboxylic acid (NH_2-TPDC) and $ZrOCl_2 \cdot 8H_2O$ in DMF in the presence of HOAc as detailed in reference [1].

2.2. Synthesis of UiO66-bpy (MOF-bipy):

The condensation reaction on UiO66-NH_2 with 2,2'-bipyridine-4-dicarboxyaldehyde (bipy-CHO) was prepared from a modified procedure from reference [4]. The UiO66-NH_2 MOF (0.037 mmol, 100 mg) was suspended in MeOH (5 mL) under ultrasound conditions for 20 minutes, and then bipy-CHO (1 mmol, 200 mg) was dissolved in MeOH (5 mL) was added, followed by the addition of acetic acid (HOAc) (25 µL). The mixture was sonicated for another 30 minutes and was heated at 70 °C for 24 h in a closed vial. The resulting precipitate was collected by centrifugation, washed with abundant MeOH (three times), and dried in air.

2.3. Synthesis of **MOF-a** (M= Re, X= Cl^-) and **MOF-b** (M=Mn, X=Br^-):

UiO66-bipyRe$(CO)_3$Cl (**MOF-a**) and UiO66-bipyMn$(CO)_3$Br (**MOF-b**) were prepared according to a previously reported procedure [5]. 100 mg of UiO66-bipy and 100 mg of $M(CO)_5Cl$ were suspended in 10 ml of toluene under sonication for 1hr and then refluxed for 3 hrs in a closed vial at 110 ^oC. The resulting precipitate was collected by centrifugation, washed with abundant dichloromethane (until the supernatant was colourless), and dried under a high vacuum.

2.4. Synthesis of **(PIB)MOF-a** (solubilization):

PIB-phosphonic acid was prepared according to a previously reported procedure [5], and the solubilized MOF was synthesized as reported previously [6]. MOF-a (50 mg) was placed in a 6 mL vial and dispersed in 2 mL of cyclohexane by sonication for 90 minutes in a closed vial purged with nitrogen. PIB1000-phosphonic acid (≈150mg) was dissolved in 1 mL of cyclohexane and was added to the reaction mixture. The reaction mixture was sonicated at 40 °C for 8 hours and stirred overnight at 40 °C in a closed vial purged with nitrogen. The reaction mixture was transferred to a centrifuge tube, and 4 mL of cyclohexane was added and then centrifuged at 3000 rpm for 15 min. The supernatant was collected to isolate the solubilized MOFs. This washing and centrifuging procedure was repeated three times. The collected supernatants were evaporated and dried under a vacuum overnight at 70 °C. The unreacted MOF nanoparticles, which were settled at the bottom of the centrifuge tube, were dried under vacuum overnight at 70 °C.

3. Figures S2 to SI7

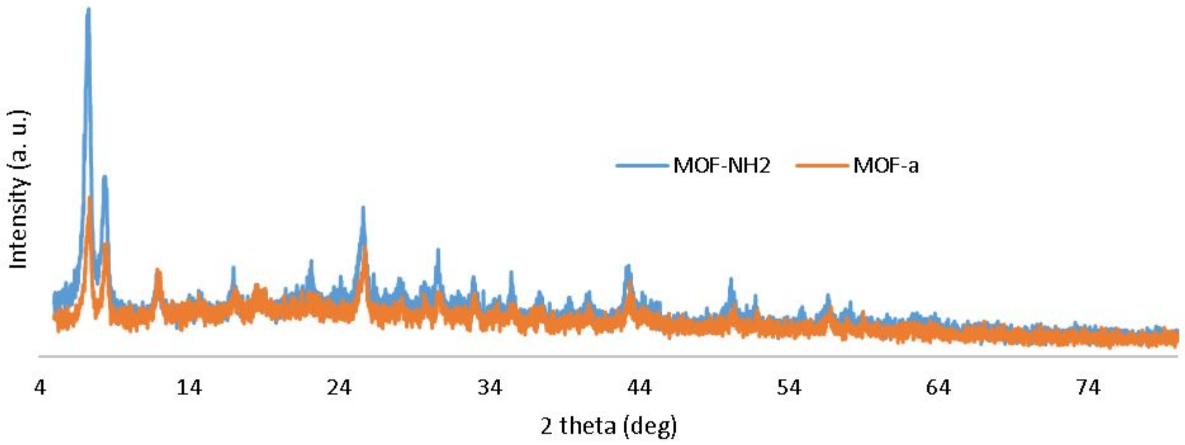

Figure S2: The results of PXRD analyses of **MOF-NH_2** and **MOF-a** verified the conservation of the crystalline structure of the materials during the condensation reaction.

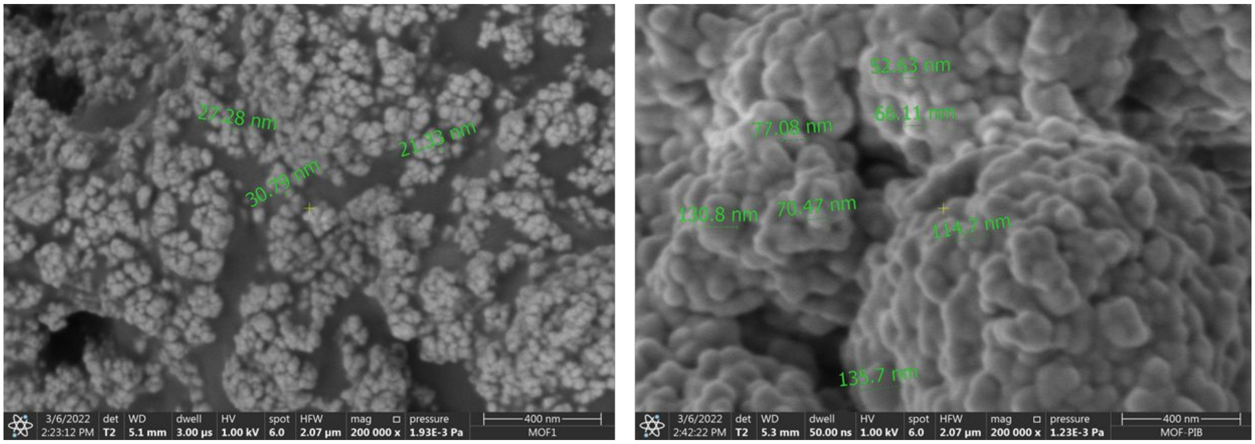

Figure S3: SEM analysis of **MOF-a** and **(PIB)MOF-a** showed nanoparticle size ranges from 20-30nm and 50-130nm, respectively. Enhancement of the size and colligation is observed as expected due to PIB.

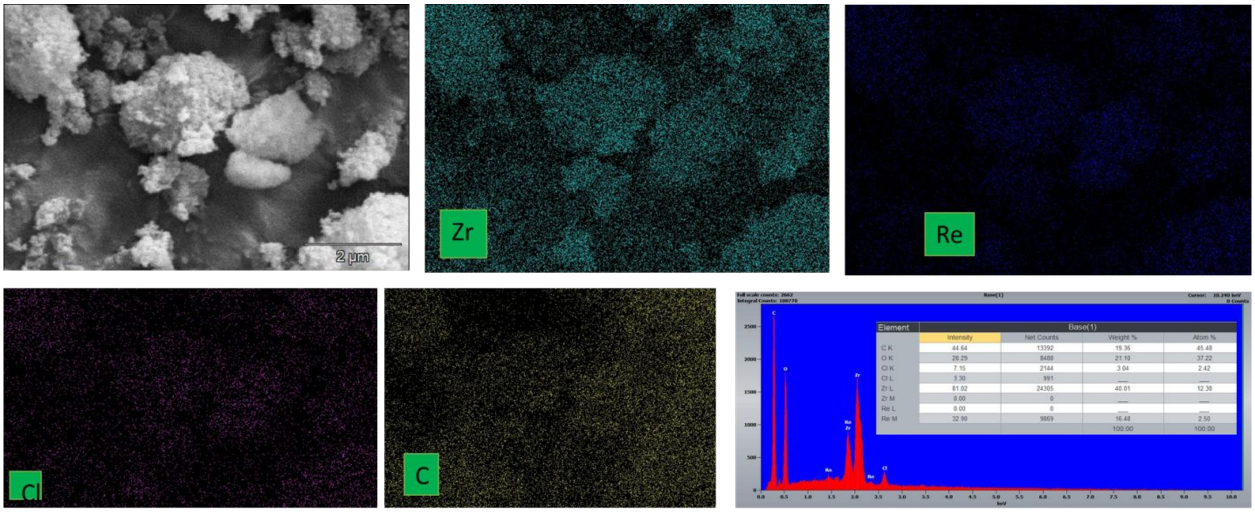

Figure S4: The EDX analysis of **MOF-a** showed a uniform distribution of Re-immobilized complexes throughout the surface of Zr-MOFs.

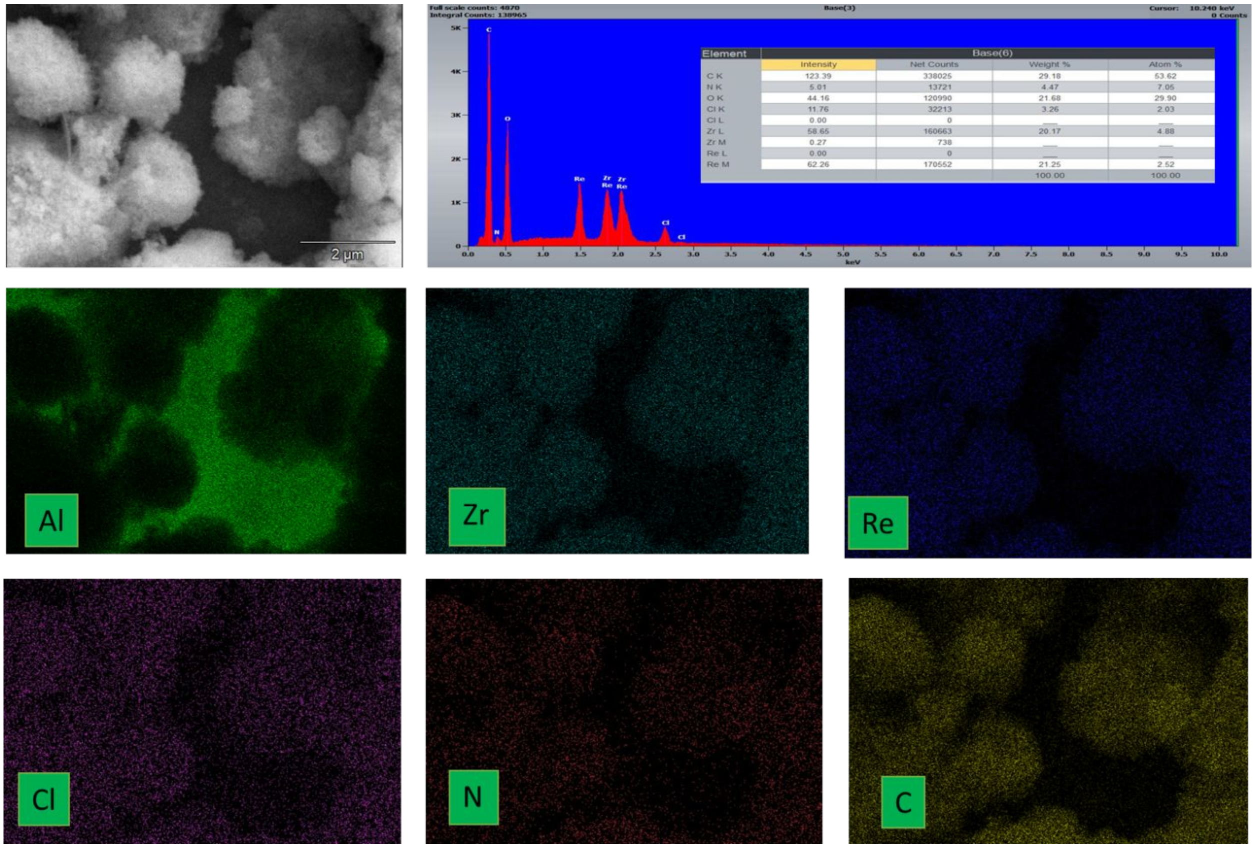

Figure S5: EDX analysis and EDX elemental mapping (Carbon, Chlorine, Nitrogen, Oxygen, Rhenium and Zirconium) for **(PIB)MOF-a** at Al sample holder.

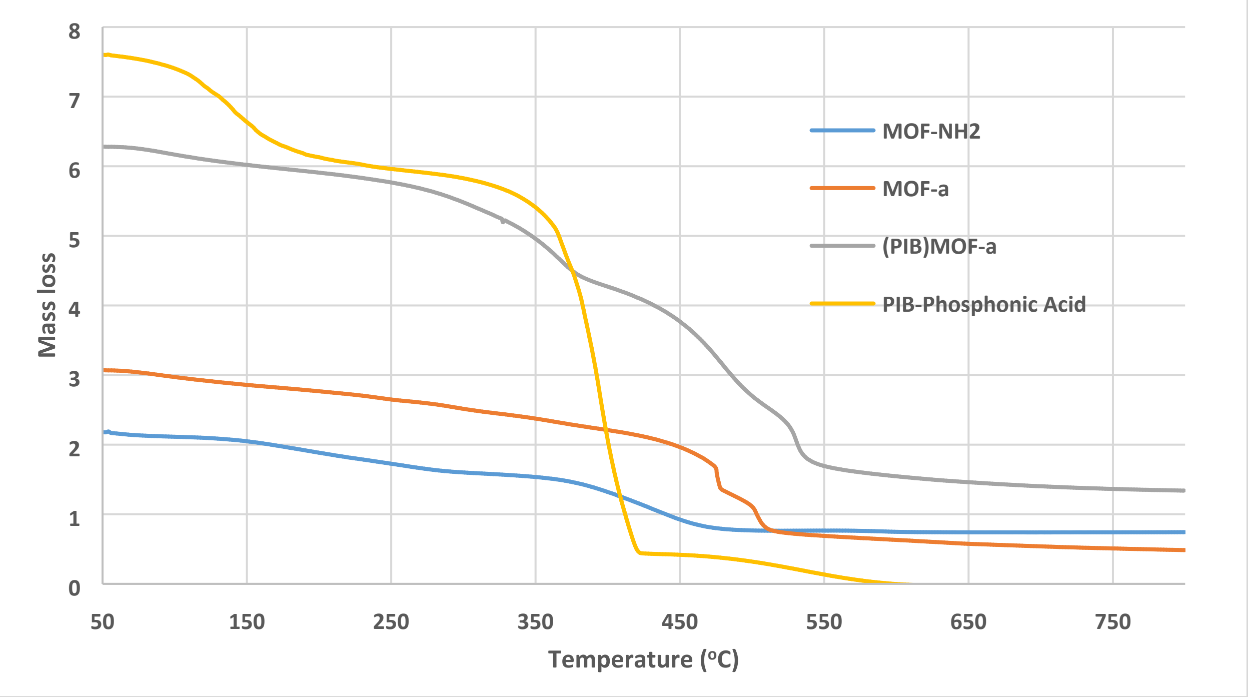

Figure S6. Comparison of TGA traces of the **MOF-NH$_2$**, **MOF-a**, **(PIB)MOF-a** and PIB-phosphonic acid, showing 64 % MOF content.

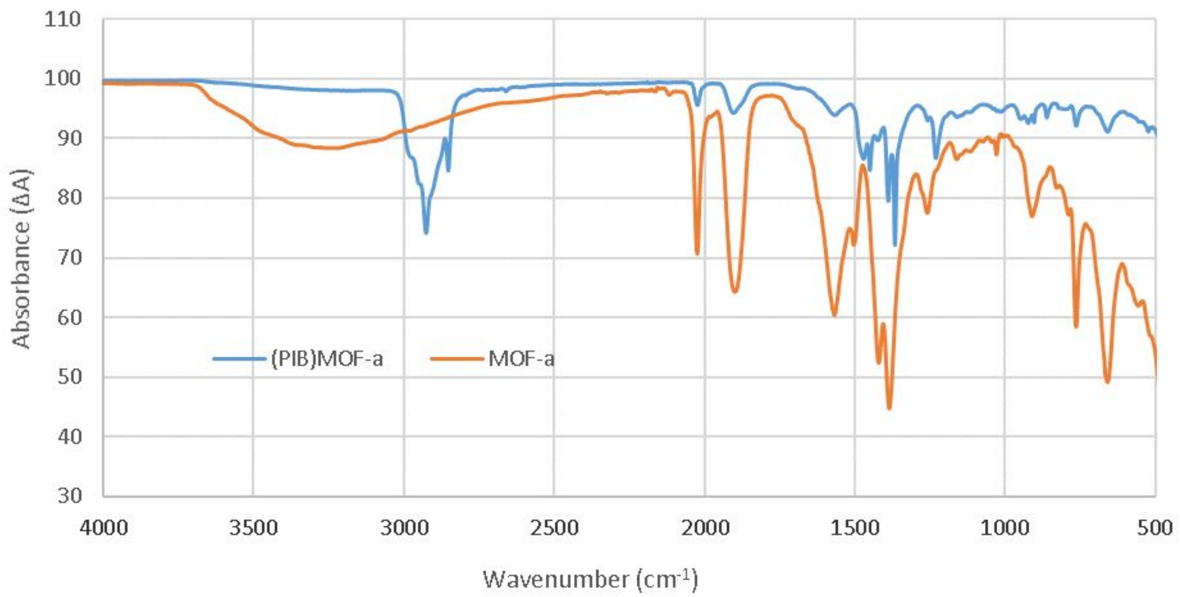

Figure S7: The solid-state IR spectrum of **MOF-a** [UiO66(bpy)Re(CO)$_3$Cl] and **(PIB)MOF-a** displayed peaks at 2031 and a broad peak at 1933 cm^{-1}.

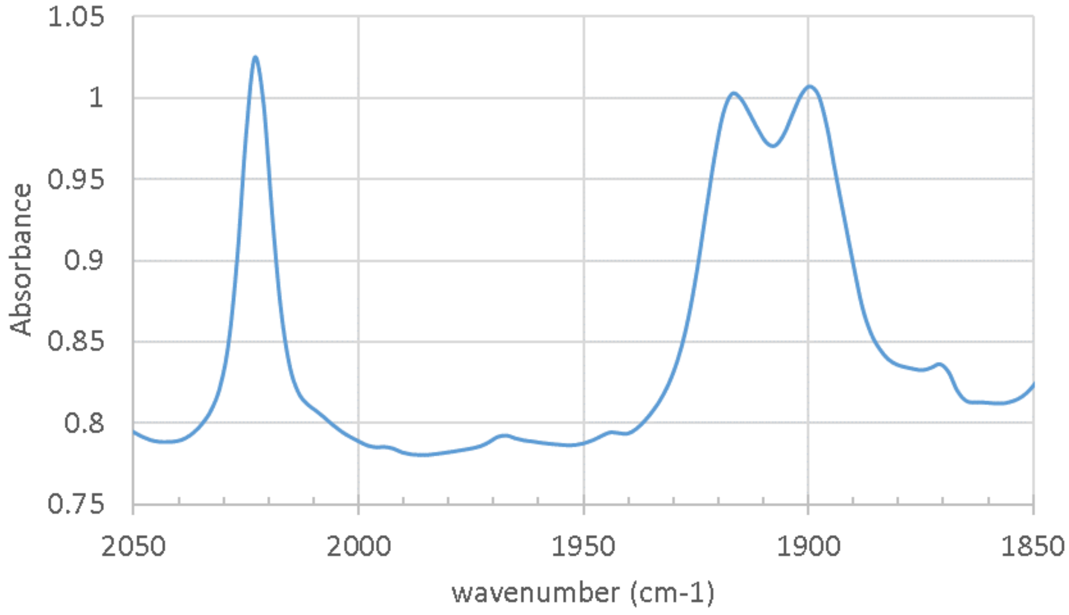

Figure S8: IR spectrum of molecular bipyRe$(CO)_3$Cl displayed peaks at 2023, 1918, and 1896 cm^{-1} in THF.

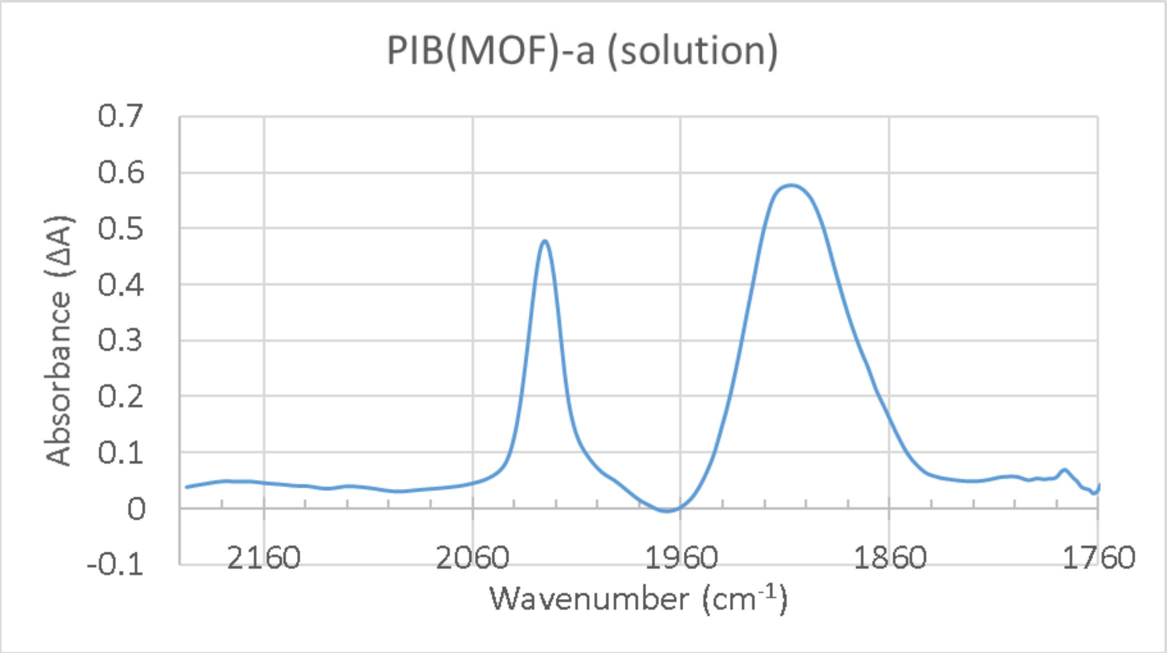

Figure S9: Solution phase IR spectrum of PIB(MOF)-a displayed peaks at 2023 and 1899 cm^{-1} (broad) in THF.

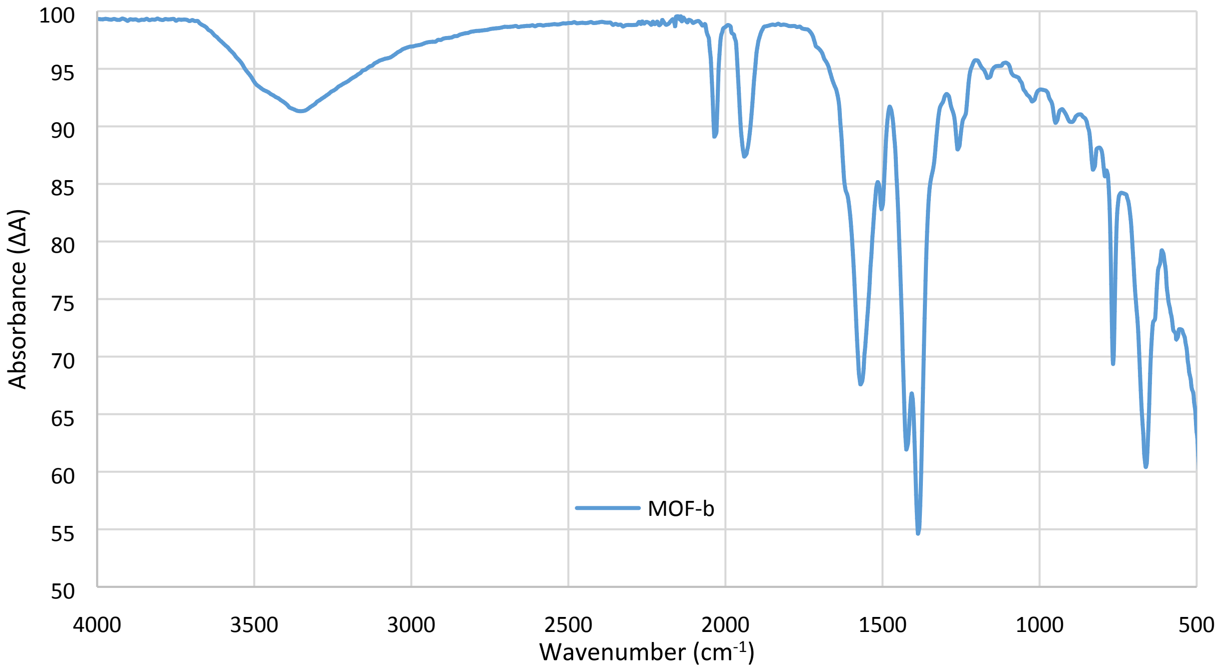

Figure S10: The solid-state IR spectrum of **MOF-b** [(UiO66-(bipy)Mn$(CO)_3$Br] displayed peaks at 2031 and a broad peak at 1933 cm^{-1}. Complete decay in peak intensity is observed during PIB immobilization and may be the result of complex decomposition.

4. References

1. Morris, W.; Briley, W. E.; Auyeung, E.; Cabezas, M. D.; Mirkin, C. A., Nucleic Acid–Metal Organic Framework (MOF) Nanoparticle Conjugates. *Journal of the American Chemical Society* **2014,** *136* (20), 7261-7264.
2. Kiefer, L. M.; Michocki, L. B.; Kubarych, K. J., Transmission Mode 2D-IR Spectroelectrochemistry of In Situ Electrocatalytic Intermediates. *The Journal of Physical Chemistry Letters* **2021,** *12* (15), 3712-3717.
3. Smieja, J. M.; Kubiak, C. P., Re(bipy-tBu)(CO)3Cl−improved Catalytic Activity for Reduction of Carbon Dioxide: IR-Spectroelectrochemical and Mechanistic Studies. *Inorganic Chemistry* **2010,** *49* (20), 9283-9289.
4. Wei, Y.-P.; Yang, S.; Wang, P.; Guo, J.-H.; Huang, J.; Sun, W.-Y., Iron (iii)-bipyridine incorporated metal–organic frameworks for photocatalytic reduction of CO 2 with improved performance. *Dalton Transactions* **2021,** *50* (1), 384-390.
5. Chao, C.-G.; Kumar, M. P.; Riaz, N.; Khanoyan, R. T.; Madrahimov, S. T.; Bergbreiter, D. E., Polyisobutylene Oligomers as Tools for Iron Oxide Nanoparticle Solubilization. *Macromolecules* **2017,** *50* (4), 1494-1502.
6. Mamlouk, H.; Elumalai, P.; Kumar, M. P.; Aidoudi, F. H.; Bengali, A. A.; Madrahimov, S. T., In Situ Solution-State Characterization of MOF-Immobilized Transition-Metal Complexes by Infrared Spectroscopy. *ACS Applied Materials \& Interfaces* **2020,** *12* (2), 3171-3178.
